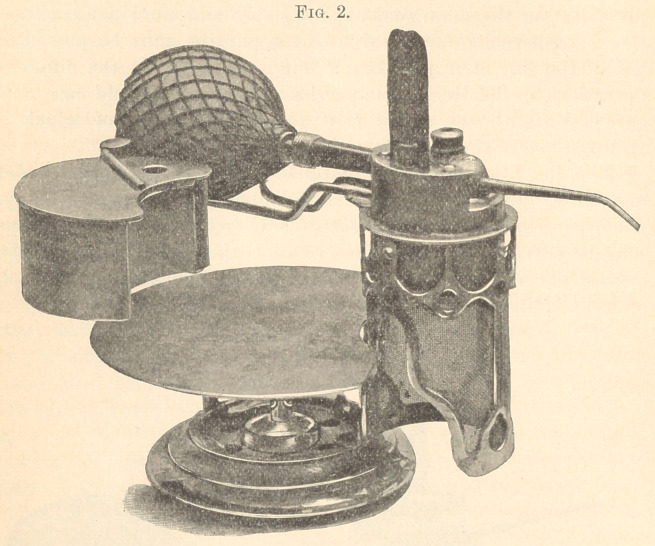# American Academy of Dental Science

**Published:** 1895-01

**Authors:** 

**Affiliations:** American Academy of Dental Science


### Reports of Society Meetings.

### AMERICAN ACADEMY OF DENTAL SCIENCE.

THE American Academy of Dental Science met with Drs. Andrews and Cutler at the Colonial Club, Quincy Street, Cambridge Massachusetts, on Wednesday evening, June 6, at 7.45 o'clock. The paper for the evening was read by Dr. James M. Daly, Professor of Operative Dentistry at the Boston Dental College. Subject, "A Method of Filling Root-Canals."

(For Dr. Daly's paper, see page 31.)

*President Smith.*—Gentlemen, this paper of Professor Daly is before you for discussion.

### DISCUSSION.

*Dr. Daly.*—Now that I am one of the Fellows I may be free to speak. I expected that some one would say that this is an old method, and a very old method. I knew it, but I wished to present it in the way of contrast to the method advocated by Dr. Shields. Perhaps I might speak of that more at length. His method of filling the root-canal was to ream out with the Donaldson nerve-canal cleanser, using some of the finest broaches with the little barbed end cut off. He says that he can thoroughly and perfectly fill every root, no matter how tortuous the canal may be, with gold.

*Dr. Williams.*—As Dr. Daly says, his is an old idea. For many years after I became tired of filling root-canals with solid gold, I used to make for myself small conical rods of zinc, of tin, and of lead for filling the roots; but these, of course, can now be obtained at the dental-supply stores. The S. S. White Dental Manufacturing Company has the tin and lead in little boxes. But I did not rely simply on the metal, I placed them in an antiseptic solution or paste before passing them up into the root, and then cut off the metal rod. As far as the "royal" metal is concerned, I should not consider it in any way superior to any other. Whatever does the most good in the therapeutical or surgical treatment of the case is the most "royal." There are some advantages over gold in using the tin or lead. They both have more conservative qualities than gold; but whatever is used, my practice has been to have it well coated with some antiseptic paste, then cutting it off, to leave a short end in the chamber of the pulp, and seal up the rest with whatever you choose, gutta-percha or any cement, but I prefer gutta-percha as a general thing. Sometimes I fill in around the rod with a paste of cement mixed with an antiseptic solution. This is particularly good for old roots, as it makes them more durable. You can leave the end of the rod long enough, so that in case of future inflammation, requiring vent of the root, you simply have to unstop the cavity and pull out the rod. No matter how sore the tooth may have been, you at once get relief and the tooth begins to mend. I was present at a meeting somewhere at which one man said that the only way to do was to fill roots so soundly with some solid material that they never would inflame afterwards. Now, that is going beyond all principles of surgery; you cannot tell what constitutional effect may come into the stump of that dead root. In talking with an old man who has lost a limb, he will tell you that for many years after it was amputated, when it was cold and damp, he would be troubled with neuralgia in that stump. So at the end of an amputated pulp, where the dead matter leaves off and the living tissues begin, there is an old scar. That may be healed up and may continue strong as long as the person is in good health, but there is still a slight weakness there from the fact that the part has not its full vitality. I have often seen cases where a person would become debilitated from some cause, or be suffering from some special depression, where an inflammation would set in and cause as much pain and trouble as if the pulp had only recently died. In such cases you could not find any reason for thinking that the root had not been antiseptically treated and filled. It is better to have a stopping which can be readily removed to give relief in case of inflammation, and not be obliged to drill and jar the inflamed tooth to remove a solid filling.

*Dr. Potter.*—To divert a little from the subject but at the same time to follow out a line suggested by the essayist, I would like to bring up the matter of arsenical preparations for discussion. It seems impossible to get a preparation of arsenic that will be absolutely painless; but is there any preparation which is more comfortable than another? I have had offered me by an agent an English preparation which was said to be painless. Of course I would not think of using it, because of its secret character, but if there are any preparations of arsenic whose composition is known which are fairly comfortable, I should like to know about them.

We often hear about "dead teeth." I am very much opposed to the use of the term "dead tooth," unless the tooth is really dead.

The term is unscientific in the majority of cases and sounds badly to a patient. If a person thinks he has a dead tooth in his mouth, he is apt to look upon it with abhorrence and doubt its usefulness. If we call such teeth "pulpless" no such unfavorable impression is given, and we are far more accurate in our statement.

*Dr. Daly.*—I trust I did not use the "dead tooth" term. I am also very much opposed to it.

In regard to arsenical preparations, there are a number of combinations which are said to be painless, but the one with which I have had the most success is one which Dr. Harlan, of Chicago, recommends. It is a mixture of arsenous acid and hydrochlorate of cocaine, equal parts, rubbed up or triturated with lanoline. It makes an easy paste to handle, and my experience has been that the application of it is without pain. I always note just how much arsenous acid is put into a preparation, that I may know what the twenty-fifth or fiftieth of a grain is.

*Dr. Fillebrown.*—If I understood Dr. Potter's question, it was not relating to the preparation which was easiest to handle, but to the one that would give the least discomfort to the patient in its application. I am inclined to think that any arsenical preparation applied to an inflamed pulp will give pain, and to make an arsenical application comfortable we must first reduce the inflammation. I never apply arsenic to a painful pulp. I invariably quiet the pain with a sedative, and often wait until another sitting before applying the arsenic. This plan produces the best results.

I object to a paste, although having used it for a number of years, because it is difficult for inexperienced persons to judge of the amount that they are putting in. They also want to be very sure that the arsenic does not come out, so they take a piece of cotton and put it into the cavity and press it home, hard and solid, and then put a large piece of gutta-percha over that. Of course that acts as a plunger and squeezes the arsenic out, and it impinges upon the membranes and produces that excruciating pain which is so vividly described in Dr. Daly's paper. In one case where necrosis followed, I found out absolutely that that was the cause. Another case resulted in a severe sloughing caused by the operator perforating the side of the root, thus allowing the paste to come in contact with peridental membrane. If there had been no pressure in either of those cases there would have been no trouble. The safest form in which it is applied at the present day is that suggested by Professor Flagg. Arsenous acid and creosote are mixed in due proportion and thoroughly diffused through some cotton fibres which have been finely chopped up. This preparation is a convenient one to handle. It is not necessary that you should put in the twenty-fifth or fiftieth of a grain; the one-hundredth of a grain will be as effectual as a grain. By first allaying the inflammation by applying sedatives, I feel that I am perfectly safe, and will be sure to give the patient no discomfort whatever, and have the best promise of success in devitalizing pulps. Gold wire for filling the apex of roots was recommended by Dr. Morrison, of St. Louis, about twenty years ago. My objection to it is the difficulty of removing it. At the present time we think it is better to be able to remove the filling and give the patient relief if trouble arises, and later, fill again. During many years of my practice it was the fashion to fill roots with gold-foil, and that was the method which I practised, and I am sure I filled curved roots. A few years since I was challenged on this matter, and I took a tooth that had a curved root and opened it up and removed the pulp, and then went on and filled it with gold-foil. I then filed through the root on the side of the curve, and, as I confidently expected, found the canal filled beyond the curve at the apex. I did it right before the eyes of the challenger, and he was quite willing to admit my success. It is generally thought now that gutta-percha and the phosphates are better. I prefer the gutta-percha, because it is difficult to carry the phosphates to the end of the root,—the residual air there is apt to form an impediment. All the gutta-percha is easily removed.

*President Smith.*—We next come to the "Incidents of Practice," and under this head will call upon Dr. Henry F. Libby, of Boston, who will exhibit a dental air-heater and annealing apparatus.

*Dr. Libby.*—In the practice of dentistry as now conducted the many operations to be performed call for apparatus of a more or less varied character; and the simplicity of such apparatus, and the rapidity with which different forms may be changed and handled, have a direct bearing upon the amount and character of the work performed by the dentist.

Accuracy is of great moment, and yet in several operations the judgment of the operator is at present the sole guide, as, for instance, in the use of the hot-air syringe or chip-blower. When it is desired to use the instrument, it is held over the flame of a lamp until the operator thinks the temperature of the heating chamber is about right, when, as a matter of fact, the temperature may be many degrees removed from that desired.

After its use the instrument cools down, and must be again heated, so that in addition to the inaccuracy described, its use involves much loss of time to both operator and patient. Again, it is frequently desirable that a gutta-percha heater or annealing-tray may be brought into temporary service, rapidly and without necessitating the arrangement of separate and more or less extensive paraphernalia, for the different apparatus must be put aside out of the way after use; but, as far as I am aware, the different operations of heating gutta-percha or annealing gold are now carried on with the aid of separate apparatus, one of which is removed before the other is brought into use.

This invention has for its object the production of a heater and annealing apparatus particularly adapted for operating rapidly, comprising various parts and features whereby different operations may be carried on without loss of time, the arrangements of the several parts being compact, simple, readily and quickly adjusted, and in condition for immediate use at all times.

Fig. 1.

Fig. 1 represents the perspective view of the apparatus as it naturally rests upon the bracket, with the reservoir in contact with the body of the heater.

Fig. 2 represents the body of the heater thrown laterally to one side, allowing the annealing-tray to be brought into position over the lamp or Bunsen burner, and the pivoted reservoir swung to one side.

Fig. 2.

The base of this device, as represented in the figures, is made quite heavy, and of sufficient size to form a firm support for the different parts.

The body frame is surrounded by wire gauze to protect the flame from draughts when in use, and to better protect its heat. In the upper part of this cylindrical framework is the sand-bath, consisting of a metallic case, and filled with sand or fine quartz.

This medium gives a uniform temperature, in which is placed the bulb of a thermometer extending into the bath; this registers the degree of heat required. In the top of this chamber is a receiver projecting into the sand, which corresponds with the elongated bulb of the hot-air syringe or chip-blower.

It is obvious that when the sand-bath is swung into position over the lamp the temperature of the bath will be accurately registered on the index of the thermometer, and the temperature of the receiver will be that of the bath, likewise that of the chip-blower, which, being placed in the receiver, will very shortly be raised to the temperature of the bath, and can in consequence be accurately determined by a glance at the thermometer.

The bulb of the chip-blower is filled with copper wire for the purpose of retaining heat, which it does sufficiently long to dry a devitalized tooth.

The temperature required for chip-blowing is 300° F. In order to provide means for heating water to different temperatures without necessitating the use of separate and cumbersome apparatus, I have secured a standard to the base, upon which is pivoted the water reservoir, made of metal, and having a metallic cover, which is provided with a small opening for the use of the water syringe.

The reservoir is shaped to correspond to the cylindrical side of the sand-bath, so that it may be swung up against the latter and rapidly heated by conduction.

The flanged portion of the water-bath resting on the annular ledge of the heater can be readily regulated by moving it towards or from the body heater. The metal top or cover of the water reservoir serves as a gutta-percha heater, and absolutely prevents any burning or overheating of the material.

In connection with this, on the outer side of the water-bath is an annular holder, which is a convenient device for holding a small vial containing an obtundent, which is also heated by its proximity to the water-bath.

The annealing-tray, as shown in Fig. 2, being swung into position over the flame, has a diameter of four and one-half inches. This is supported by an upright standard secured to the base.

William H. Potter, D.M.D.,
*Editor American Academy of Dental Science.*

## Figures and Tables

**Fig. 1. f1:**
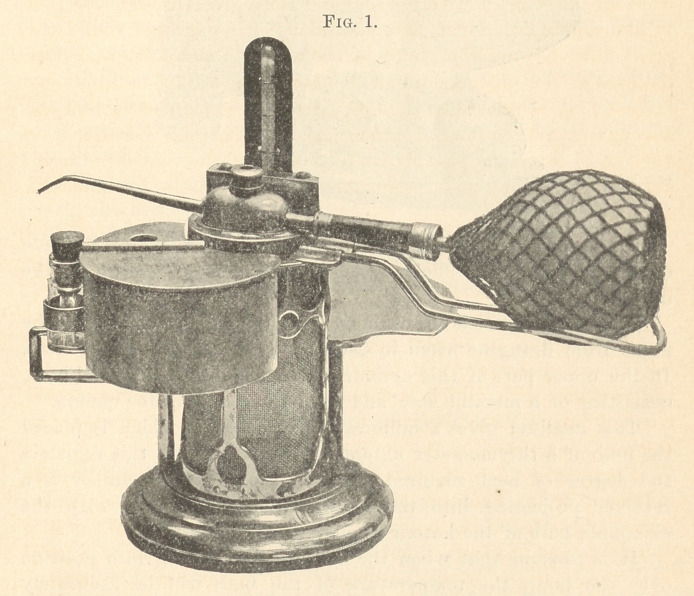


**Fig. 2. f2:**